# An Emerging Role for SNARE Proteins in Dendritic Cell Function

**DOI:** 10.3389/fimmu.2015.00133

**Published:** 2015-03-31

**Authors:** Laura E. Collins, Joseph DeCourcey, Mariana Soledad di Luca, Keith D. Rochfort, Christine E. Loscher

**Affiliations:** ^1^Immunomodulation Research Group, School of Biotechnology, Dublin City University, Dublin, Ireland

**Keywords:** dendritic cells, SNAREs, IL-6, IL-23, IL-12

## Abstract

Dendritic cells (DCs) provide an essential link between innate and adaptive immunity. At the site of infection, antigens recognized by DCs via pattern-recognition receptors, such as Toll-like receptors (TLRs), initiate a specific immune response. Depending on the nature of the antigen, DCs secrete distinct cytokines with which they orchestrate homeostasis and pathogen clearance. Dysregulation of this process can lead to unnecessary inflammation, which can result in a plethora of inflammatory diseases. Therefore, the secretion of cytokines from DCs is tightly regulated and this regulation is facilitated by highly conserved trafficking protein families. These proteins control the transport of vesicles from the Golgi complex to the cell surface and between organelles. In this review, we will discuss the role of soluble *n*-ethylmaleimide-sensitive factor attachment protein receptor proteins (SNAREs) in DCs, both as facilitators of secretion and as useful tools to determine the pathways of secretion through their definite locations within the cells and inherent specificity in opposing binding partners on vesicles and target membranes. The role of SNAREs in DC function may present an opportunity to explore these proteins as novel targets in inflammatory disease.

## Introduction

Dendritic cells (DCs) were first described by Ralph Steinman and Zanvil Cohn as a rare murine splenocyte with dendrite-like protrusions that displayed phagocytic abilities (1). Following their discovery, work carried out by Steinman’s group demonstrated that DCs play a vital role in orchestrating immunity. In their immature state in the peripheral tissue, DCs are characterized by a high capacity for antigen capture and processing via phagocytosis. They display distinct chemokine receptor expression, chemokine responsiveness, and low T cell stimulatory capabilities (2). The ability of DCs to regulate immunity is dependent on their maturation, which is induced by the interaction of DCs with antigens that are subsequently presented to T cells. During this process, DCs secrete distinct profiles of cytokines and chemokines and express co-stimulatory molecules required to drive specific T cell responses (3). The control of these secretory pathways is an essential factor in regulating innate and adaptive immune homeostasis and controlling responses to infection (4). In this review, we will discuss the factors that induce DC maturation, the cytokines secreted by DCs that drive adaptive immunity, and the secretory pathways that have been identified and their potential as immunotherapeutic targets.

## Dendritic Cell Activation

For maturation to occur, foreign antigens must be identified by DCs. DCs express pattern recognition receptors (PRRs) that allow them to recognize microbes that display pathogen-associated molecular patterns (PAMPs). PRRs can be divided into toll-like receptors (TLRs), C-type lectin receptors (CLRs), nucleotide-binding domain and leucine-rich repeat containing receptors (NLRs) and retinoic acid-inducible gene-I (RIG)-like receptors (RLRs) (5). Following engagement of these PRRs, signaling cascades are activated that induce the production of inflammatory cytokines (6). Inflammatory responses can also be triggered by endogenous damage-associated molecular patterns (DAMPs). These are intracellular markers that are also recognized by PRRs, and are released during tissue damage or when a cell is necrotic releasing intracellular material into its surroundings (7). Following PRR ligation and subsequent maturation, DCs become antigen presenting cells (APC) (8). The mature DC travels to the local lymph node (9), which facilitates the presentation of antigen to antigen-specific lymphocytes to prime naive T cells to become effector T cells. Three signals are required for this: antigen presentation, up-regulation of co-stimulatory markers, and secretion of cytokines; this review will focus on the third signal, cytokine secretion and their effects (10).

## Cytokine Polarization of T Cell Subsets

Clonal immunity involves DC activation of T cells through presentation of processed antigen to naïve T cells in an immunogenic form and the secretion of immune mediators called cytokines. Cytokines are classified into interleukins (IL), interferons (IFNs), and colony-stimulating factors. Cytokine profiles secreted from DCs polarize naïve T cells into different phenotypes of CD4^+^ T cells. These differential cytokine secretion patterns are dependent on the PRR activation, and subsequently are the key signals required for the differentiation into different T helper (Th) cell phenotype subsets that are required to clear the antigen that trigger the immune response (11).

In order to clear bacterial and viral infections, naïve CD4^+^ T-cells require cytokine signaling from DCs to initiate differentiation into a Th1 phenotype. TLRs present on DCs recognize PAMPs, such as lipopolysaccharide and unmethylated dsDNA, through TLR4 and TLR9 respectively. Engagement of these TLRs results in secretion of the cytokines IL-1, IL-6, TNF-α, and IL-12p40 (10). Dimerization of IL-12p40 with IL-12p35 results in an active IL-12p70 heterodimer. IL-12p70 together with IFN-γ, which is produced in large amounts by activated T-cells, leads to differentiation of T-cells in to an IFN-γ producing Th1 cells subset (12). Th2 cells produce high levels of IL-4, IL-5, and IL-13. They are responsible for initiating the humoral immune response and are required to clear helminth infections. Th2 cells have been generated *in vitro* via stimulation of the TCR and the addition of IL-4 (13). IL-4 induces the induction of Th2 cells via STAT6, given the fact that DCs do not produce IL-4, the mechanisms through which Th2 cells initiate this subset remain unclear. However, cytokine signaling from DCs can indirectly affect the differentiation. DCs activated through TLR2, DC-SIGN, and OSCAR (an FcγR-associated receptor) lead to the activation of Erk1 and Erk2 pathways and phosphorylation of c-Fos (14). This leads to the inhibition of IL-12 and increased production of IL-10 in DCs, which is sufficient to induce a strong Th2 response in naïve T-cells. Cytokines produced by other cells can also influence DCs to drive a Th2 subset through chemokine production that recruits IL-4 producing cells and the activation of cytokine receptors (11).

Th17 cells were first described in 2006 and identified as a T helper subset distinct from Th1 and Th2 cells that produced the cytokine IL-17 (15). Th17 cells have been indicated to confer protection against extracellular bacteria and fungi by the secretion of effector molecules such as IL-17, IL-21, IL-22, GM-CSF, and CCL20 (16). However, Th17 cells have also been implicated in many autoimmune and inflammatory diseases, such as rheumatoid arthritis (RA), systemic lupus erythematosus, multiple sclerosis, psoriasis, and inflammatory bowel disease (IBD) (17). The conditions for differentiation of Th17 cells is more complex than Th1 and Th2 subsets, and there is evidence to support a role for a number of cytokines produced by DCs, including IL-1, IL-18, IL-6, and IL-23. These cytokines are produced by DCs in response to PRR recognition of bacteria and fungi.

In the case of Th17 differentiation, it is not just TLR-mediated activation that promotes the secretion of these cytokines. Dectin-1 and dectin-2 activate CLRs on DCs to promote section of IL-6 and IL-23, cytokines that play a major role in differentiating and stabilizing the Th17 response respectively (18). T-regulatory cells are essential for negative regulation of the immune response, as they possess immunosuppressive abilities that confer protection to the host from autoimmunity and prevention of excessive immunopathology. If this response becomes dysregulated, it can lead to autoimmune disorders due to loss of homeostasis. IL-27, IL-10, and TGF-β1 secretion by DCs have been heavily indicated in the induction of Treg cells (19).

## Targeting Cytokines in Inflammatory Disease

Cytokines are essential in driving the adaptive immune response; thus, targeting cytokines associated with inflammatory disease has become a significant area for drug development (20). Indeed, secretory pathways are attractive to study as potential targets to block critical cytokines with the possibility of switching off inflammation without any detrimental side effects (21). A number of therapeutics currently exist that target key cytokines such as IL-23 and IL-6, which are associated with inflammatory disease.

IL-23, essential for stabilizing Th17 cells, has been implicated in the pathogenesis of multiple sclerosis, arthritis, and IBD. Using mouse models of diseases, the essential role of IL-23 has been established (16, 22, 23). A human monoclonal antibody, Ustekinumab, has been developed and licensed to target the IL-12/23 pathway. This antibody targets the p40 subunit of IL-12 and IL-23, and was originally developed to target IL-12 induction of Th1 cells, as IL-23 had still not yet been identified or recognized in its function of driving Th17 differentiation. These applications include psoriatic arthritis, multiple sclerosis, and Crohn’s disease, and the drug has displayed good efficacy except in the case of multiple sclerosis (24).

IL-6 has also been implicated as an important cytokine in driving the Th17 subset. Aberrant expression of IL-6 has been indicated in inflammatory diseases such as RA and juvenile idiopathic arthritis (JIA), leading to the development of a humanized IL-6 receptor (25). Tocilizumab blocks IL-6 receptor in both soluble and membrane bound forms with the result of blocking IL-6 function. In the clinic, tocilizumab has demonstrated amelioration of inflammatory diseases such as RA and JIA with improvement in symptoms such as joint destruction and a reduction in the number of flare incidents in JIA (26).

## Secretion of Immune Mediators

It is clear that DC cytokine release is essential and tailored to clearing specific infections while also controlling immune homeostasis. Regulation of protein trafficking and release of immune mediators such as cytokines, chemokines, and lysosomal enzymes into the immediate environment from DCs is therefore highly regulated in order to prevent autoimmunity and inflammation. Indeed, a number of mutations or deficiencies resulting in dysregulated trafficking can have major implications in immune syndromes (27).

Depending on the required function of an immune mediator, different pathways exist to transport newly synthesized proteins out of the cell. These pathways are usually shared by a number of factors and their release can be constant (constitutive release) or triggered (regulated release) (4). Proteins produced in the endoplasmic reticulum (ER) can be soluble and contained within vesicles or membrane bound and associated within the plasma membrane of transported vesicles.

Thus, a number of proteins share transport to the plasma membrane to conserve energy (27). The release of these proteins can be basal or dependent on their transcription at gene level in response to cellular signaling (28).

## Snare Proteins

The transport of vesicles between organelles, endosomes, and the cell surface plasma membrane is controlled by a complex network of cellular machinery of membrane associated proteins, lipids, and cytoplasmic proteins. Soluble-N-ethylmaleimide–sensitive factor accessory protein receptor proteins (SNAREs) are a large conserved family identified in the docking/fusion machinery that allow membranes to overcome opposing forces and fuse docked lipid bilayers (27).

SNARE proteins are all identified by a common conserved SNARE motif of 60–70 amino acids arranged in heptad repeats typical for coiled coils (29). Most SNAREs are type II integral membrane proteins that possess a single trans-membrane domain at their carboxyl (C-terminal) end that is connected to the SNARE motif by a flexible linker, with the majority of the protein extending into the cytoplasm (30). The conserved coiled-coil SNARE motif is necessary for complex formation and membrane fusion (31). When SNAREs form in a tight helical bundle, they form a conserved structure of an elongated coiled coil of four intertwined α-helices that correspond to the four interacting SNARE motifs occupying specific positions in the complex (32). There is a highly conserved layer of interacting amino acids in the central hydrophobic core of the helical bundle consisting of three glutamines and one arginine (31). Each of these contributing motifs contributes an amino acid and their structures are divided into sub-families used to further classify the SNARES as R-SNAREs or Q-SNARES (33).

R-SNARES are usually found on the membrane and have arginine (R) as the central functional residue in the SNARE motif. Q-SNARES are mainly found on the target membrane and are defined by a central glutamine (Q) residue. In order to form a complex and mediate membrane fusion, one R-SNARE and two or three Q-SNARE motifs are required to form a stable RQabc four helical bundle (30, 34). The SNARE proteins on opposing membranes interact to form the α-helix bundle that “zippers” together from the N-terminus to the C-terminus forming a trans-SNARE complex (or SNAREpin). The energy provided by the zippering complex formation pulls the membranes together displacing the water and hydrostatic pressure keeping them apart, thereby facilitating the mixing of hydrophobic lipids (35). Once the membranes have fused, the trans-SNARE conformation is converted to a highly stable inert cis-SNARE conformation on the resulting fused membrane. In order to dissociate this stable structure, metabolic energy is required and this is provided by two chaperone proteins; *N*-ethylmaleimide-sensitive factor (NSF), and Soluble NSF attachment protein (SNAP) (36, 37). This dissociation facilitates the recycling of SNARE proteins and prevents accumulation of SNAREs on the membrane. Many SNAREs operate predominantly in a specific subcellular compartment with their partners and some Qa SNAREs such as Syntaxin (STX)2 and STX4 are found on the plasma membrane and involved in fusion of vesicles and secretion of their contents from the cell. Other Qa SNAREs including STX5 and the R-SNARE vesicle-associated membrane protein (VAMP)4 are localized in the Golgi apparatus (38). These SNAREs are therefore found in the membranes of the donor compartment, acceptor compartment, and the intermediate trafficking vesicles (39).

## The Role of SNAREs in Dendritic Cells

As the immune system consists of a plethora of cells that have specific functions, an understanding of the mechanism of trafficking is extremely important for when these functions become dysregulated and lead to disease. Cytokine signaling from DCs, in particular, is one of the most important types of signaling for the regulation of the adaptive immune response. However, while the role of SNAREs in secretion of key immune mediators has been addressed in many immune cells, the role of SNAREs in DCs function is poorly understood. Examples of SNAREs regulating critical function in other immune cells include STX11 which has been implicated in neutrophil degranulation and interferon-γ (IFN-γ) secretion from natural killer cells (40). Inhibition of SNAP23 or STX3 abolished chemokine release from mast cells and has been attributed to STX11. (41). While these individual SNAREs and their function have been identified in these cell types, a full elucidation of these trafficking pathways and/or SNARE partners have yet to be identified. However, in the last 10 years, Stow and colleagues have mapped the secretion of TNF-α from macrophages and fully elucidated an original secretion pathway through recycling endosomes that is implicitly linked to their phagocytic activity (42–44).

Furthermore, loss of STX11 in macrophages resulted in increased phagocytosis and TNF-α secretion, suggesting that this SNARE plays its part as a negative regulator (45). This hypothesis is supported in patients with the hyper-inflammatory disease, familial hemophagocytic lymphohistiocytosis type 4 (FHL-4), which results from a deletion or a mutation in STX11 (46). These patients possess high levels of pro-inflammatory cytokines (IFN-γ, IL-6, TNF-α, and IL-18) and have a higher number of macrophages that become over-activated and have increased phagocytic activity (47, 48). From their findings in macrophage, Offenhäuser et al. (45) accredited STX11’s regulatory ability due to it how it binds and inhibits other SNARE proteins via its conserved SNARE sequence and lack of a trans-membrane domain (45). A role for STX11 in regulation of DCs has also been suggested. STX11 was examined in Bone marrow-derived dendritic cells (BMDCs) by D’Orlando et al. (40), and it was reported that although mRNA levels were up-regulated in BMDCs, STX11 deficiency did not affect BMDC activities or TLR-induced secretion of IL-12, IL-6, or TNF-α (41). Work by our own group, in a paper published in this issue of *Frontiers*, shows a correlation of up-regulation of STX11 expression in BMDCs in response to TLR ligands. Further work is required to confirm the role of STX11 in DCs although it is possible that the role of STX11 in phagocytic APCs is conserved (49).

VAMP8 is an R-SNARE that has also been reported to significantly inhibit phagocytosis in DCs (50). In this study, the authors show that VAMP8 associates with lysosome-associated membrane protein-2 (LAMP2) at the phagosome in dendritic cells, following bacterial exposure. Use of *VAMP8*^-/-^ DCs significantly increased phagocytic ability. Furthermore, over-expression of VAMP8 in a DC2.4 cell line inhibited phagocytosis. Ho et al. also suggest that VAMP7 also negatively regulates phagocytosis in the same manner, as both VAMP7 and VAMP8 can form SNARE complexes with STX7, STX8, and Vti1b (50). This complex has been show to mediate homotypic fusion of early and late endosomes (51).

SNARE expression in DC has been reported in some detail in a paper describing differential expression following treatment with acetylsalicylic acid (ASA), the active ingredient in aspirin. Cai et al. reported that SNARE expression of Vti1a, Vti1b, VAMP3, VAMP8, and STX8 were all up-regulated following treatment with ASA (52). The authors describe a decrease in phagocytosis in response to ASA, and attribute it to the up-regulation of Vti1a and Vti1b on phagosomes, and inhibition of SNARE complex formation. This study also describes the up-regulation of VAMP8 in cells that have decreased phagocytic ability, corroborating the findings by Ho et al. (50). Vti1b can also form a SNARE complex with VAMP 8 in the previously described STX7/STX8/Vti1b SNARE complex identified on endosomal membranes (51).

**Figure 1:**
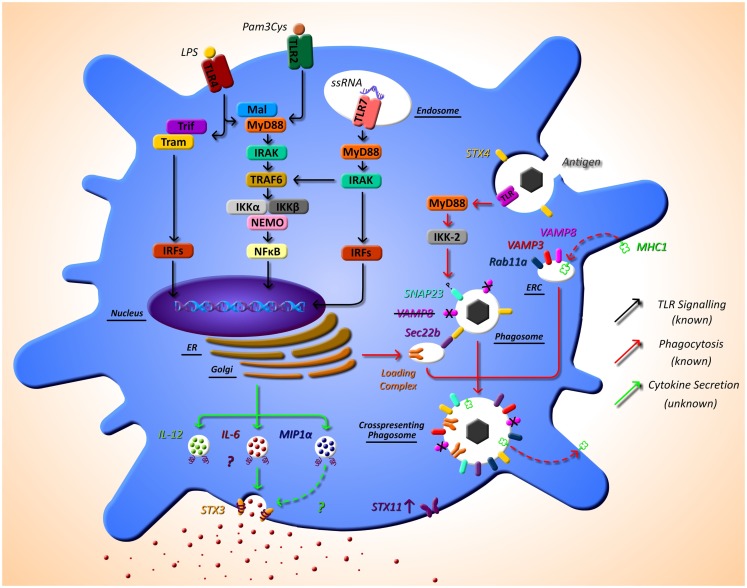
**The as yet unknown side of DC secretion post-TLR activation, and trafficking molecules currently identified in DC function**. Following TLR activation with TLR ligands, such as LPS, DCs activate intracellular pathways that lead to the transcription of inflammatory mediators, such as cytokines. While this pathway is extremely well defined (black arrows), very little is known of the post-transcriptional pathway and how proteins, in particular cytokines, are trafficked out of the DC. It is evident from other cell types that SNARE proteins play a role in secretion of cytokines; however, to date, their specific role has not yet been described. SNARE importance in DCs has been highlighted by studies that demonstrate that STX4 on the phagosome interacts with Sec22b on the ER-Golgi intermediate compartment, which is required for cross-presentation and MyD88-dependent TLR signaling, and results in phosphorylation of SNAP23 present on the phagosome. This leads to fusion with endosomal recycling compartment (ERC) and ultimately cross-presentation. Furthermore, VAMP8 has been shown to be a negative regulator of phagocytosis (red arrows). Our recent work has indicated a role for STX3 in IL-6 and possibly MIP1-α secretion (green arrows). This work and others also suggests a role for STX11 in DC function as it is highly up-regulated post TLR2, TLR4, and TLR7 activation.

Cross-presentation is another essential function of DCs that is regulated by SNARE proteins. Sec22b was identified by Cebrian et al. (2011) as a specific regulator of cross-presentation. Sec22b is localized to the ER-Golgi intermediate compartment which interacts with STX4 on the phagosome. In the absence of Sec22b, DCs were unable to transfer antigen from phagosomes to the cytosol and degradation of the antigen in the phagosome increased (53). It was recently shown that while Sec22b-mediated phagosomal recruitment is required for cross presentation, it is independent of TLR signaling and cannot deliver MHC class I (MHC-I). Nair-Gupta et al. have shown that large quantities of MHC-I are stored in the endosomal recycling compartment (ERC) (which possess Rab11a, VAMP3, and VAMP8 on its surface). TLR signaling through the myeloid differentiation (MyD) marker, MyD88, results in phosphorylation of SNAP-23 present on the phagosome. This leads to stabilization of SNARE complexes, fusion of ERC and the phagosome, and ultimately cross-presentation (54). These findings regarding cross-presentation are summarized in Figure 1.

Work in our own group is currently identifying the mechanisms of secretion of key immune mediators involved in inflammatory disease and we have recently identified a role for STX4 in IgE secretion from plasma cells (55). Due to the role of DCs in the direction of adaptive immune responses via secretion of cytokines, we aim to elucidate these pathways in detail in these cells. Another finding from the research paper published in this issue of *Frontiers*, we have described the effects of different TLR ligands on DCs and measured the cytokine profiles secreted by these cells and their concurrent SNARE expression (49). Specific TLR pathway activation allows DCs to respond as needed to specific challenges. For example, when TLR4 is activated by lipopolysaccharide (LPS), this in turn activates DCs to produce the cytokines necessary to orchestrate an adaptive immune response to clear a bacterial infection, as described above. Depending on the TLR agonist stimulation, different cytokine profiles are secreted by DC, and we demonstrate that these profiles correlate to differential expression of SNAREs. Assessing the secretion of cytokines from JAWSII DC’s following activation with TLR ligands revealed that IL-6 and MIP-1α secretion was significantly increased following stimulation with LPS (TLR4) and Loxoribrine (TLR7) but not PGN (TLR2). This correlated with a change in expression of STX3. Furthermore, knockdown of STX3 using siRNA against STX3 resulted in a significant decrease in IL-6 and MIP-1α secretion with no effect on IL-1β or TNF-α, indicating a role for STX3 in IL-6 secretion. To our knowledge, this is the first study to profile SNARE expression patterns following different TLR stimulations in DCs and demonstrates the correlation between cytokine secretion patterns and expression of SNAREs (49). This is overviewed in Figure 1, indicated by the green arrows.

## Conclusion

Identifying specific SNARE complexes and their regulators will lead to a better understanding of DC function during inflammation and may present potential therapeutic targets in a wide range of inflammatory diseases. Current therapeutic monoclonal antibodies target aberrant and subsequent damaging cytokine production by blocking their activities. It is well known that prevention is better than a cure and the current therapeutic strategies do not meet this requirement, highlighting the need for more targeted therapies. Monoclonal antibodies treatments are also prone to off-target effects such as drug induced liver damage and the generation of anti-idiotypic antibodies, rendering them ineffective (56). While a number of combined antibody therapies and personalized medicine methodologies are being investigated, targeting trafficking proteins and pathways to inhibit cytokine release from the cell may prove to be a more effective therapeutic approach.

## Conflict of Interest Statement

The authors declare that the research was conducted in the absence of any commercial or financial relationships that could be construed as a potential conflict of interest.
